# Revisiting the Mangled Extremity Severity Score (MESS) in Popliteal Artery Injury: A Single-Centre Experience in Vietnam

**DOI:** 10.7759/cureus.38813

**Published:** 2023-05-10

**Authors:** Lam Van Nut, Huynh Thanh Son, Nguyen Lam Vuong

**Affiliations:** 1 Department of Vascular Surgery, Cho Ray Hospital, Ho Chi Minh City, VNM; 2 Department of Medical Statistics and Informatics, Faculty of Public Health, University of Medicine and Pharmacy at Ho Chi Minh City, Ho Chi Minh City, VNM

**Keywords:** prediction, mangled extremity severity score, injury, trauma, popliteal artery

## Abstract

Background: Popliteal artery injury is a severe condition that can lead to limb loss. Early intervention is essential to achieve optimal outcomes, including limb salvage. The Mangled Extremity Severity Score (MESS) is a scoring system used to predict amputation rates for mangled limb injuries. The effectiveness of the MESS in predicting amputation in patients with traumatic popliteal artery injury is unclear, particularly in settings with a high prevalence of motorcycle accidents.

Methods: This retrospective study was conducted at a single center in Vietnam between January 2018 and June 2020. The study included 120 patients who underwent surgical treatment for popliteal artery injury. Data were collected from electronic medical records, radiology reports, and operative notes. Logistic regression model and the area under the curve (AUC) were used to evaluate the predictive value of the MESS.

Results: Patients with a MESS score of ≥8 had a higher rate of amputation compared to those with a MESS score of <8. However, the predictive value of the MESS was limited, with an AUC of 0.68. Higher skeletal/soft tissue injury score, limb ischemia score, and shock score were associated with a higher risk of amputation. The age score of the MESS was unexpectedly higher in the limb salvage group.

Conclusions: The MESS score can be useful in predicting amputation rates in patients with popliteal artery injury, but its predictive value is limited. A team approach involving experienced surgeons is recommended for decision-making regarding amputation.

## Introduction

Popliteal artery injury is a complex and potentially devastating condition that can lead to limb loss, especially when not diagnosed and treated promptly. It is one of the most common types of vascular injury, accounting for up to 20% of peripheral vascular injuries [1]. The severity of popliteal artery injury varies widely, ranging from simple contusions to complete transections with significant ischaemia. Management of a popliteal artery injury involves a multidisciplinary approach, with various treatment options available, including conservative management, surgical repair, or endovascular intervention. Early intervention is crucial to achieving optimal outcomes, including limb salvage.

In recent years, several scoring systems have been implemented to grade the severity of trauma and predict the need for primary amputation in mangled extremities. Among them, the Mangled Extremity Severity Score (MESS) has been widely used to guide the decision-making process for the treatment of popliteal artery injury. The MESS includes information about skeletal and soft tissue injury, limb ischaemia, shock, and age of the patient [2]. A MESS score of 7 or higher has been defined as a predictor for the need for primary amputation [2]. However, there is still controversy over the use of the MESS in predicting limb salvage and primary amputation rates in civilian trauma [3,4]. Several studies have reported low predictive value for limb salvage as well as primary amputation using the MESS [5,6]. Others have reported the potential of the scoring system to identify patients at risk for primary amputation [7,8]. The discrepancies in the findings may be attributed to differences in the study population, the definition of limb salvage, and the scoring system used.

Despite its limitations, the MESS remains an important tool in the decision-making process for the treatment of popliteal artery injury. In a systematic review published in 2015, the MESS was found to be the most commonly used scoring system for grading the severity of trauma [9]. However, the same review highlighted the need for further studies to evaluate the effectiveness of the MESS in predicting primary amputation rates in different patient populations.

In our country, a significant number of people experience traumatic popliteal artery injuries resulting from motorcycle-related traffic accidents. To our knowledge, there is no existing study on the application of MESS in managing popliteal artery injuries in this particular setting. Therefore, the aim of this study is to evaluate the use of the MESS as a predictor of amputation rates in patients with traumatic popliteal artery injury. We hope that the findings of this study will contribute to the body of knowledge on the management of popliteal artery injury and provide useful information for clinicians in the decision-making process for the treatment of these complex injuries.

## Materials and methods

Study design and population

This study is a retrospective analysis of patients who underwent surgical treatment for traumatic popliteal artery injuries at the Department of Vascular Surgery, Cho Ray Hospital, a tertiary care hospital in Ho Chi Minh City, Vietnam, between January 2018 and June 2020. Ethical approval for this study was obtained from the Ethics Committee of the University of Medicine and Pharmacy at Ho Chi Minh City (No. 312/HDDD-DHYD, dated 8 May 2020).

Data collection

Data were collected from electronic medical records, radiology reports, and operative notes. Demographic data, including age, sex, and comorbidities, were collected. Details on the mechanism of injury, type of vascular trauma (dissection, thrombosis or transection), the anatomical level of the popliteal artery injury (above or below the knee), and type of arterial reconstruction were recorded. The severity of the trauma was graded for each patient before surgery using the MESS. The MESS was the total score of four components: the skeletal/soft tissue injury, limb ischaemia, shock, and age. If the ischaemia time was greater than six hours, its score was doubled. Concomitant skeletal, venous, and neural injuries were also documented, as well as details on fasciotomy (primary or secondary, open or subcutaneous). The length of hospital stay and in-hospital amputation rates were also assessed.

Outcome measurement

The primary outcome of interest was limb salvage or amputation. Amputation was decided intraoperatively by orthopaedic and vascular surgeons. All patients underwent open surgery as soon as possible after the diagnosis of their popliteal artery injury. However, the surgery was delayed in some cases, such as overloading of patients or the occurrence of shock that required resuscitation before the surgery. During the surgery, the tibial muscles were checked first. There are four anatomical compartments: the anterior, lateral, superficial posterior, and deep posterior compartments. If all muscles in three compartments or more were determined as necrosis, amputation was performed. Otherwise, the necrotic muscles were excised, and the popliteal artery was re-anastomosed. In some patients whose limb was not improved after revascularization, they underwent another surgery after three days to evaluate the muscles in all compartments. If more muscles were necrotic, a delayed amputation was performed.

Statistical analysis

Patient characteristics were summarised using mean ± standard deviation or median (25th; 75th percentiles) for numerical variables and frequency (percentage) for categorical variables. The descriptive analysis was performed for all patients and in two groups: MESS of <8 and ≥8. The cut-off of 8 was chosen based on previous studies that suggested this threshold was appropriate [10]. Differences between the two groups were tested using the Mann-Whitney U test for numerical variables and Fisher's exact test for categorical variables. To evaluate the predictive value of MESS on the outcome of amputation, a logistic regression model was performed with a single covariate, MESS. The predicted probability of amputation based on MESS was visualised. The area under the receiver operator characteristic (ROC) curve (AUC), sensitivity, specificity, positive predictive value (PPV), and negative predictive value (NPV) of each MESS value were also derived. The Youden index (Youden index = sensitivity + specificity - 1) was used to define the best cut-off. The best cut-off was the one with the highest Youden index value. All analyses were performed using R statistical software version 4.1.0 (R Foundation for Statistical Computing, Vienna, Austria).

## Results

From January 2018 to June 2020, 120 patients underwent surgical treatment for popliteal artery injury in our hospital. The minimum, median, and maximum MESS on admission were 3, 8, and 12, respectively. There were 51 patients with a MESS of <8 and 69 patients with a MESS of ≥8.

Patient characteristics are shown in Table 1. The mean age was 32.9 years and males predominated (80.8%). Motor vehicle accidents were the main cause of trauma (94.2%). Twenty-three patients (19.2%) had shock on admission. In 100 patients (83.3%), the limb ischemia time was more than six hours. The group with a MESS of ≥8 had a higher mean age, a higher percentage of shock at admission, and a longer time of limb ischemia, which is explained by the components of the MESS. In 49 patients (40.8%), the operation was performed late (>12 hours after the trauma). Most patients (93.3%) had concomitant bone injuries; 13 (10.8%) had multiple injuries, and five (4.2%) had a concomitant neural injury. Primary amputation was performed in 11 patients (9.2%), and secondary amputation was performed in two other patients. The median length of hospital stay was five days and was longer in the group with a MESS of ≥8.

**Table 1:** Patient characteristics Statistics are mean ± standard deviation, median (25th; 75th percentiles) or n (%). @There were 11 patients undergoing primary amputation without popliteal revascularization because all muscles in three or more anatomical compartments were necrotic. Two other patients underwent amputation three days after popliteal revascularization. MESS, Mangled Extremity Severity Score

	Overall (N=120)	MESS < 8 (N=51)	MESS ≥ 8 (N=69)	p-value
Age, years	32.9 ± 11.9	30.1 ± 9.8	34.9 ± 13.0	0.063
Sex				0.816
Female	23 (19.2)	9 (17.6)	14 (20.3)	
Male	97 (80.8)	42 (82.4)	55 (79.7)	
Mechanism of trauma				0.236
Motor vehicle accident	113 (94.2)	50 (98.0)	63 (91.3)	
Work-related accident	7 (5.8)	1 (2.0)	6 (8.7)	
Shock at admission	23 (19.2)	1 (2.0)	22 (31.9)	<0.001
Limb ischemia				<0.001
Reduced pulse but normal perfusion	17 (14.2)	17 (33.3)	0 (0.0)	
Pulseless, slow capillary refill	56 (46.7)	29 (56.9)	27 (39.1)	
Cool, paralysis, numb/insensate	47 (39.2)	5 (9.8)	42 (60.9)	
Ischemia time > 6h	100 (83.3)	38 (74.5)	62 (89.9)	0.045
Delayed operation (> 12 h after trauma)	49 (40.8)	18 (35.3)	31 (44.9)	0.349
Concomitant bone injury	112 (93.3)	49 (96.1)	63 (91.3)	0.464
Concomitant neural injury	5 (4.2)	3 (5.9)	2 (2.9)	0.649
Multiple injuries	13 (10.8)	7 (13.7)	6 (8.7)	0.392
Primary amputation^@^	11 (9.2)	2 (3.9)	9 (13.0)	0.114
Amputation	13 (10.8)	3 (5.9)	10 (14.5)	0.152
Liver impairment	51 (42.5)	17 (33.3)	34 (49.3)	0.095
Renal impairment	23 (19.2)	7 (13.7)	16 (23.2)	0.244
Hyperkalemia	23 (19.2)	13 (25.5)	10 (14.5)	0.161
Length of hospital stay (days)	5.0 (4.0; 6.0)	4.0 (3.5; 6.0)	5.0 (4.0; 7.0)	0.043

Apart from 11 patients with primary amputation, in 109 patients who underwent popliteal revascularization, the mean operating time was 3.4 hours. Fasciotomy was performed in all patients. Most popliteal injuries occurred below the knee (87.2%), and the main type of injury was dissection or thrombosis (73.4%). In 56 cases (51.4%), a direct reanastomosis was performed. For the others (48.6%), an interposition graft was used because the length of the injury was too long for a direct reanastomosis. Seven patients (6.4%) required a re-intervention of the artery (Table 2).

**Table 2:** Operative characteristics of patients undergoing popliteal revascularization Statistics are mean ± standard deviation or n (%). MESS, Mangled Extremity Severity Score

	Overall (N=109)	MESS < 8 (N=49)	MESS ≥ 8 (N=60)	p-value
Operation duration (hours)	3.4 ± 1.2	3.5 ± 1.4	3.3 ± 1.1	0.409
Fasciotomy	109 (100.0)	49 (100.0)	60 (100.0)	
Popliteal segment				1
Below the knee	95 (87.2)	43 (87.8)	52 (86.7)	
Above the knee	14 (12.8)	6 (12.2)	8 (13.3)	
Vascular injury type				0.394
Dissection/thrombosis	80 (73.4)	38 (77.6)	42 (70.0)	
Transection	29 (26.6)	11 (22.4)	18 (30.0)	
Concomitant venous injury	14 (12.8)	4 (8.2)	10 (16.7)	0.253
Revascularization method				0.445
Direct reanastomosis	56 (51.4)	23 (46.9)	33 (55.0)	
Interposition graft	53 (48.6)	26 (53.1)	27 (45.0)	
Vascular re-intervention	7 (6.4)	5 (10.2)	2 (3.3)	0.240

**Figure 1:**
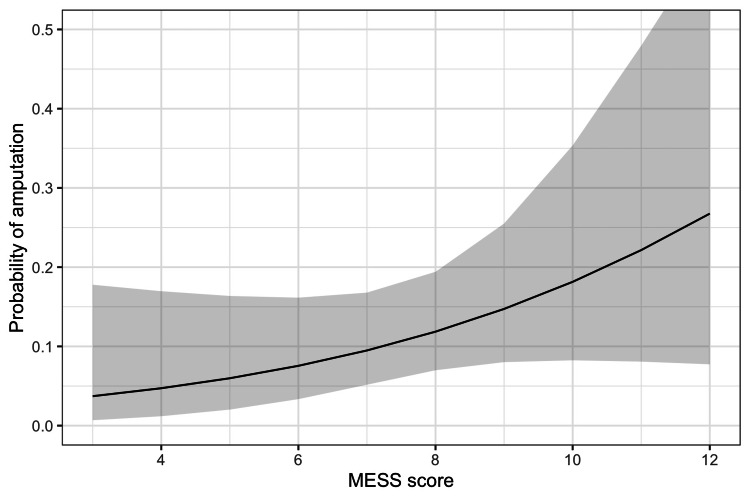
Prediction of amputation using MESS score The prediction is estimated from a logistic regression model of amputation with a single covariate MESS. The black line represents the predicted probability of amputation. The grey region represents 95% confidence interval of the estimates. MESS, Mangled Extremity Severity Score

**Figure 2:**
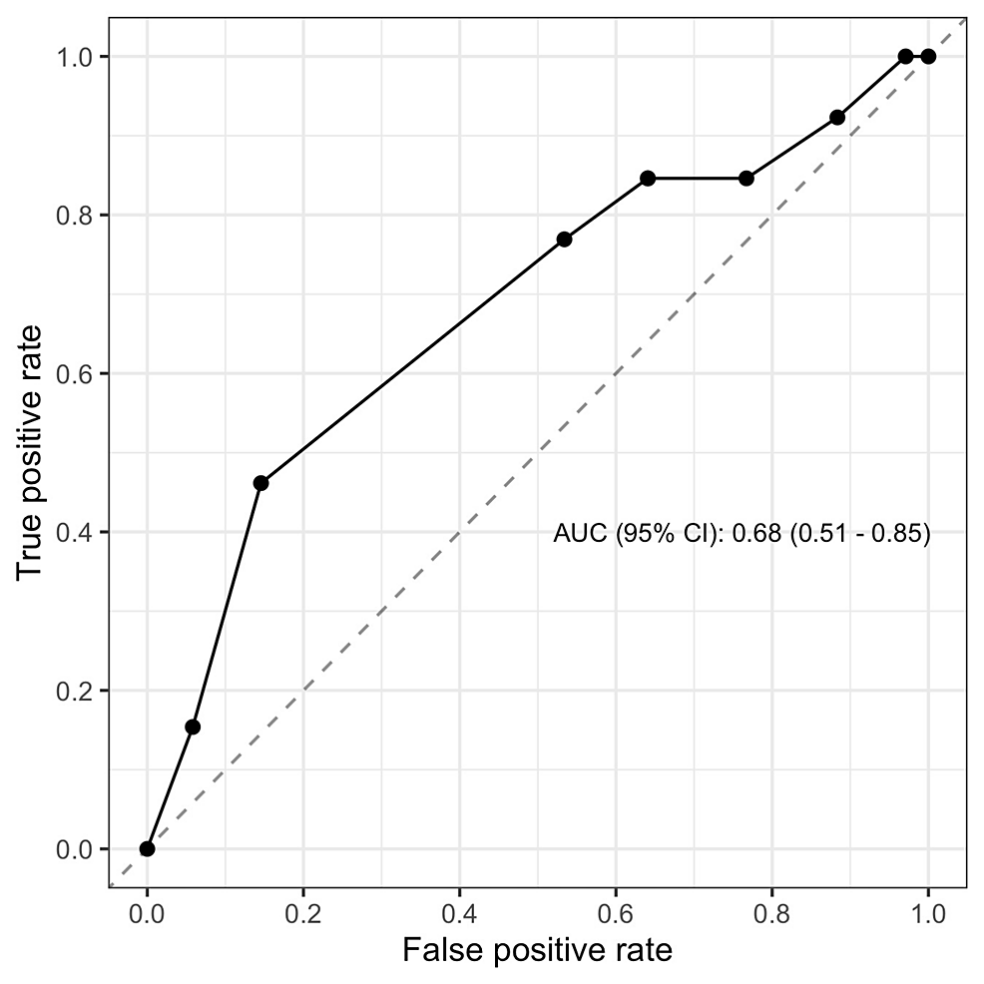
ROC curve of MESS score to predict amputation AUC, area under the curve; CI, confidence interval; MESS, Mangled Extremity Severity Score; ROC, receiver operating characteristic

In Table 3, we compared the MESS and its components between the limb salvage and amputation groups. The amputation group had a higher skeletal/soft tissue injury score, limb ischemia score, shock score, and total MESS, without significant difference. However, the age score was higher in the limb salvage group, which is not as expected. Overall, the higher MESS was associated with a higher risk of amputation (Figure 1). The predictive value of MESS was not very good. The AUC (95% confidence interval) was 0.68 (0.51-0.85) (Figure 2). The best cut-off based on Youden index was 8 with a sensitivity of 0.462 and a specificity of 0.822 (Table 4).

**Table 3:** MESS components by limb salvage and amputation Statistics are mean ± standard deviation or n (%). MESS, Mangled Extremity Severity Score

	Limb salvage (N=107)	Amputation (N=13)	p-value
Skeletal/soft tissue injury score	2.2 ± 0.9	2.8 ± 1.2	0.068
Limb ischemia score	4.1 ± 1.5	4.6 ± 1.6	0.384
Shock score	0.2 ± 0.4	0.3 ± 0.8	0.921
Age score	0.7 ± 0.6	0.2 ± 0.4	0.022
Total MESS	7.2 ± 1.9	8.0 ± 1.8	0.063
MESS ≥ 8	59 (55.1)	10 (76.9)	0.152

**Table 4:** Predictive values of MESS score for amputation MESS, Mangled Extremity Severity Score; NPV, negative predictive value; PPV, negative predictive value

MESS	Sensitivity	Specificity	PPV	NPV	Youden index
3	1.000	0.028	0.111	1.000	0.028
4	0.923	0.112	0.112	0.923	0.035
5	0.846	0.224	0.117	0.923	0.070
6	0.846	0.346	0.136	0.949	0.192
7	0.769	0.449	0.145	0.941	0.218
8	0.462	0.822	0.240	0.926	0.284
9	0.154	0.907	0.167	0.898	0.061
10	0.000	0.963	0.000	0.888	-0.037
11	0.000	0.991	0.000	0.891	-0.009

## Discussion

Popliteal artery injury is a complex and potentially devastating condition that can result in limb loss. Early intervention is critical for achieving optimal outcomes, including limb salvage. The aim of our study was to assess the utility of the MESS as a predictor of amputation rates in patients with traumatic popliteal artery injury. Our results indicate that the MESS score was associated with a higher risk of amputation in these patients. Specifically, patients with a MESS score of ≥ 8 had a higher rate of amputation compared to those with a MESS score of < 8. However, the predictive value of the MESS was only moderate, with an AUC of 0.68.

Our study also revealed that certain components of the MESS, including higher skeletal/soft tissue injury score, limb ischemia score, and shock score, were associated with a higher risk of amputation. These results are expected since higher scores in these categories represent more severe injury, more severe limb ischemia, and delayed surgery due to shock. However, we did observe an unexpected higher age score in the limb salvage group. This could be due to the fact that younger patients were more likely to have a more severe accident, given that most patients in our study sustained their injury in a motorbike accident. To the best of our knowledge, this study is the first to report on a large series of popliteal artery injuries in a single center in Vietnam. While previous studies with larger sample sizes or registry data from national trauma databases have been published, these reports reflect trauma distribution in European or US settings, where patients are mainly affected by gun-related trauma, sport-related accidents, work-related accidents, or accidents from other types of vehicles [5,11-13]. Our data better reflect trauma distribution and the use of MESS in the Vietnamese setting.

Despite the usefulness of the MESS score, we concur with other authors that it requires revision [10,14]. Our study, as well as others, suggests that the predictive value of the MESS is limited. In addition, the age component of the MESS appears to be contradictory in our setting, indicating that this component should not be used. Nonetheless, the MESS still reflects the severity of patients, and higher scores were associated with a higher risk of amputation. The cut-off value of 8 is still the most effective in predicting amputation in patients with popliteal artery injury. In our setting, we propose a team approach to decision-making regarding amputation, rather than relying solely on the MESS score. Experienced surgeons from trauma, orthopedic, and vascular disciplines should evaluate the patient at the bedside and during the surgery to make better decisions. Continued postoperative re-evaluation is also crucial in saving the limb, as two patients in our cohort required amputation even after revascularization.

Our study has several limitations. Firstly, it is a retrospective study conducted at a single center, which may limit the generalizability of our findings. Secondly, the sample size was relatively small, which may have affected the statistical power of our analysis. Lastly, we did not evaluate long-term limb function or patient quality of life. The association between the MESS and long-term outcomes is unknown.

## Conclusions

Our study suggests that the MESS score is useful in predicting amputation rates in patients with popliteal artery injury, but its predictive value is limited. Other factors, such as the severity of associated injuries and intraoperative evaluation by experienced surgeons, should also be taken into account in the decision-making process for the treatment of these complex injuries. Further studies are needed to evaluate the effectiveness of the MESS score and other scoring systems in predicting outcomes in different patient populations.
